# Internet access is a necessity: a latent class analysis of COVID-19 related challenges and the role of technology use among rural community residents

**DOI:** 10.1186/s12889-022-13254-1

**Published:** 2022-04-27

**Authors:** Sarah J. Dow-Fleisner, Cherisse L. Seaton, Eric Li, Katrina Plamondon, Nelly Oelke, Donna Kurtz, Charlotte Jones, Leanne M. Currie, Barb Pesut, Khalad Hasan, Kathy L. Rush

**Affiliations:** 1 School of Social Work and Centre for the Study of Services to Children and Families, University of British Columbia Okanagan, Kelowna, BC V1V 1V7 Canada; 2 School of Nursing, University of British Columbia Okanagan, Kelowna, BC V1V 1V7 Canada; 3 Faculty of Management and Principal’s Research Chair (Tier 2) in Social Innovation for Health Equity and Food Security, University of British Columbia Okanagan, Kelowna, BC V1V 1V7 Canada; 4 Rural Coordination Centre of British Columbia, Vancouver, Canada; 5 Department of Community Health Sciences, Cummings School of Medicine, University of Calgary, Calgary, Canada; 6 Faculty of Medicine, University of British Columbia Okanagan, Kelowna, BC V1V 1V7 Canada; 7 School of Nursing, University of British Columbia, Vancouver, BC V6T 2B5 Canada; 8 Computer Science, University of British Columbia Okanagan, Kelowna, BC V1V 1V7 Canada

**Keywords:** Technology, COVID-19, Challenges, Internet use, Latent class analysis

## Abstract

**Background:**

Rural and remote communities faced unique access challenges to essential services such as healthcare and highspeed infrastructure pre-COVID, which have been amplified by the pandemic. This study examined patterns of COVID-related challenges and the use of technology among rural-living individuals during the first wave of the COVID-19 pandemic.

**Methods:**

A sample of 279 rural residents completed an online survey about the impact of COVID-related challenges and the role of technology use. Latent class analysis was used to generate subgroups reflecting the patterns of COVID-related challenges. Differences in group membership were examined based on age, gender, education, race/ethnicity, and living situation. Finally, thematic analysis of open-ended qualitative responses was conducted to further contextualize the challenges experienced by rural-living residents.

**Results:**

Four distinct COVID challenge impact subgroups were identified: 1) Social challenges (35%), 2) Social and Health challenges (31%), 3) Social and Financial challenges (14%), and 4) Social, Health, Financial, and Daily Living challenges (19%). Older adults were more likely to be in the Social challenges or Social and Health challenges groups as compared to young adults who were more likely to be in the Social, Health, Financial, and Daily Living challenges group. Additionally, although participants were using technology more frequently during the COVID-19 pandemic to address challenges, they were also reporting issues with quality and connectivity as a significant barrier.

**Conclusions:**

These analyses found four different patterns of impact related to social, health, financial, and daily living challenges in the context of COVID. Social needs were evident across the four groups; however, we also found nearly 1 in 5 rural-living individuals were impacted by an array of challenges. Access to reliable internet and devices has the potential to support individuals to manage these challenges.

**Supplementary Information:**

The online version contains supplementary material available at 10.1186/s12889-022-13254-1.

## Background

COVID-19 was declared a pandemic and global health event by the World Health Organization on March 11, 2020 [1]. The pandemic, and response to it, has had far-reaching effects on many spheres of life worldwide. Globally, the economy has been impacted [2], with significant interruptions to supply chains for many products [3, 4]. Grocery supply chains had to respond to increasing demand as consumers prepared more meals at home following restaurant closures and the demand for grocery delivery and pickup services increased [5]. Additionally, physical distancing measures to mitigate the spread of the virus impacted mental health worldwide with high levels of both depression (24%) and anxiety (21.3%) globally [6]. In healthcare, non-urgent procedures were postponed and non-COVID visits decreased, resulting in unmet health needs and delayed care [7, 8]. Finally, the pandemic led to a greater reliance on technology to access healthcare services, connect socially, and maintain access to basic daily needs [9, 10].

With much of healthcare and technology being centralized in urban areas, rural and remote communities continued to face disparities in access to essential services. Systemic differences or disparities in health and health outcomes, known as health inequities, are caused by the unfair distribution of resources, wealth, ongoing colonialism and structural racism, and power within and between societies (Commission of the Pan American Health Organization on Equity and Inequalities in the Americas, 2019). Prior to the COVID-19 pandemic, rural-urban health disparities were well documented [11]. Rural and remote living Canadians were already disproportionately affected by environmental, social, and economic factors, such as limited access to healthcare, education and income opportunities, and food security [12]. Rural residents also face greater challenges related to COVID-19, as they are on average older, more likely to have underlying health conditions, and have less access to healthcare [13]. The pandemic amplified existing inequities, particularly for Indigenous Peoples, women experiencing domestic violence, people with disabilities, people needing medical treatment, the elderly, and people in need of housing or facing food insecurity [14]. Rurality is yet another factor contributing to amplified inequities during the pandemic.

Indeed, the pandemic forced many activities of daily life to move to online modalities [15], exacerbating the well-known rural-urban digital divide in Canada [16]. Shortly after the pandemic started (July 2020), urban internet speeds increased nearly 25 megabits per second (Mbps), while rural internet speeds plateaued at 5.5 Mbps [17]. As public health safety measures for the pandemic focused on encouraging people to go online for work, information, essential services (e.g., food, shopping, healthcare), and social connections, rural communities faced significant barriers compared to their urban counterparts. The lack of equitable internet speed meant this shift was more difficult among rural communities. Given these challenges, coupled with limited access to the internet, the COVID-19 pandemic has likely increased the burden of health inequity although the extent is unknown. Thus, it is important to better understand the multifaceted challenges that rural-living community members are facing during COVID-19, and to explore the role of technology use related to those challenges. Gaining such an understanding will provide the basis for better addressing the needs and health inequities in rural-living communities.

The aims of this study were to examine the impact of COVID-19 related challenges among rural community members and explore differences based on sociodemographic characteristics, as well as to examine the use of technology related to these challenges.

## Methods

### Study design, setting, and recruitment

This study used a cross-sectional online survey with both quantitative and qualitative questions related to the impact of COVID-19 related challenges and the use of technology. Participants were eligible to complete the survey if they were 19 years of age or older and were living in a community in a Western Canadian province considered to be rural or remote (i.e., outside the commuting distance of a larger centre with population greater than 12,000).^1^ Three quarters of the region is mountainous and the geographic area includes forests, lakes, grass plains and deserts along with 40,000 islands [18]. Online surveys were completed between May 29, 2020 and July 8, 2020. This survey immediately followed the first wave of the COVID-19 pandemic in the province. During the time of the survey, the province was in initial stages of re-opening (first provincial re-start began mid-May 2020) [19, 20]; however, immediately prior to the survey several restrictions were in place beginning March 2020: Non-essential services, dine-in restaurants, and parks/playgrounds were closed; non-urgent surgeries were postponed; non essential travel was restricted; and schools were closed and children and youth were learning from home [21].

Recruitment targeted the interior region of the province, though participation was open to all rural community members in the province. Recruitment involved social media posts on Kijiji (Canadian Craig’s list), Facebook, and Twitter, rural community association newsletters, advertisements on rural websites, word of mouth, and emails sent through researchers’ community networks. Social media posts targeted local rural community pages (e.g., “Everything [community name]”) together totally over 35,000 members and were shared through rural association social media feeds, as well as through 2 paid Facebook advertisements (“post boosts”) targeting adults living within a 25 miles radius of several rural communities in the interior region of the province. Although we were unable to track how many potential respondents were reached in total, Kijiji ads were viewed by 21 participants, and the two Facebook advertisements had a combined estimated audience reach of over 7400 adults. Three $100 and one $400 draw prize incentives were offered to promote participation. All participants provided informed consent online prior to completing the survey. Ethics approval was received from The University of British Columbia – Okanagan Behavioural Research Ethics Board. All methods were carried out in accordance with relevant guidelines and regulations.

### Measures

The online survey included items related to the impact of COVID-related challenges experienced, technology use and challenges, and sociodemographic characteristics. The survey included both Likert-type questions and open-ended responses. See supplemental materials for all survey questions used in this study (Additional file 1).

#### COVID-related challenges

A list of 12 challenges was generated based on the Canadian Pandemic Influenza Preparedness Guidelines [12]. The challenges were related to the impact of limitations in four areas: social, healthcare, financial, and daily living needs in the context of the COVID-19 pandemic. Participants rated the impact of each COVID-19 challenge on a 5-point Likert scale (1 = not at all; 5 = extremely). Cronbach’s alpha for the challenges scale was 0.85. For analyses, items were dichotomized to reflect low impact (0 = not at all, very little, somewhat) and high impact (1 = quite a lot, extremely). Participants were also invited to provide an open-ended response to the question “Can you please tell us about the most significant challenge you have faced during the COVID-19 pandemic?”

#### Technology access and use, positive experiences, and challenges

Drawn from the 2018 Canadian Internet Use Survey [23] and Statista Research Department [24], participants were asked if they had access to internet in their home and if they had enough connected devices to meet their needs. Frequency of technology use was measured by asking participants how often technology was used to connect with others and to gather information prior to and during COVID-19. Participants were also invited to provide open-ended responses to the following questions: “What has been positive about your experience using technology during the COVID-19 pandemic?” and “What has been your biggest challenge around using technology during the COVID-19 pandemic?”

#### Sociodemographic characteristics

Finally, the survey included self-reported demographic items related to age, gender, ethnicity/race, occupation, and education level. See Table 1 for full break down of demographic characteristics. In addition, information about living situation (i.e., type of home, rent or own, and number of individuals co-habiting) and participant location was obtained. Lastly, participants were asked if they identified as a person with a disability and about their general health status. Self-reported sociodemographic data were aggregated based on the variability observed in responses. Due to lack of variability in some responses, ethnicity/race variable was categorized into three groups: Indigenous (e.g., First Nation, Métis, Multiracial with Indigenous heritage), Caucasian only (e.g., no other background identified), and all other responses (e.g., Asian, African). Education was coded into three categories: at least some high school, a college/trade certification, and university degree. Finally, gender-based analyses were conducted for males and females, as there were fewer than 5 non-binary/gender fluid respondents.

**Table 1:** Characteristics of study population

Sample Characteristics	***n***	%
Age (years)		
19–35	57	20.4%
36–54	95	34.1%
55+	103	36.9%
Prefer not to answer/Missing	24	8.6%
Gender		
Male	72	25.8%
Female	197	70.6%
Other/Preferred not to answer/Missing	10	3.6%
Race/Ethnicity		
Caucasian	210	75.3%
Indigenous	36	12.9%
Other	27	9.7%
Prefer not to answer/Missing	6	2.2%
Disability		
Yes	37	13.3%
No	232	83.1%
Prefer not to answer/Missing	10	3.6%
Education		
At least some high school	53	19.0%
Trades certification/diploma	124	44.4%
University degree	101	36.2
Missing	1	0.4%
Number of children (0–18) in the home		
None	50	17.9%
1	32	11.5%
2	32	11.5%
3 or more	20	7.2%
Missing	145	51.9%
Number of adults (19–64) in the home		
None	36	12.9%
1	118	42.3%
2	45	16.1%
3 or more	21	7.5%
Missing	59	21.2%
Number of older adults (65+) in the home		
None	49	17.6%
1	58	20.8%
2	12	4.3%
Missing	160	57.3%
Home type		
Single-family home	168	60.2%
Home on a farm/ranch	65	23.3%
Multifamily home (apartment, townhouse, condo)	46	16.5%
Rent or Own Home		
Rent	65	23.3%
Own	204	73.1%
Missing	10	3.6%

### Analytic approach

#### Descriptive statistics

Descriptive statistics (frequencies and means/SDs) were used to summarize the data. Initial descriptive and bivariate analyses were also conducted to examine the distribution of the challenge items and to ensure that the items were statistically, as well as conceptually, related.

#### Latent class analysis

Latent class analysis (LCA) was used to generate classes, or groups, based on similar patterns of COVID-related impacts across 12 challenges. LCA is a finite mixture-modeling approach designed to detect latent classes, or groups, of individuals based on a pattern of similar responses across a set of categorical indicators [25]. Using full information maximum likelihood with robust standard error, this approach can handle missing data as part of the response pattern. Thus, individuals were only excluded from analyses if data were missing on all 12 indicators (*n* = 1).

LCA models using the dichotomized 12 challenge items were estimated for up to a 5-class solution. Fit was determined using three comparative fit indices: Akaike information criterion (AIC), Bayesian information criterion (BIC), and sample size adjusted Bayesian information criterion (ABIC), as well as the bootstrap likelihood ratio (BLRT). Lower AIC, BIC, and ABIC suggests better fit and a non-significance likelihood-ratio test indicates that model with an additional class (e.g., 4 vs. 5 classes) does not offer a better fit [26, 27]. We also examined entropy, which indicates the overall accuracy of classification, with a value closer to 1 meaning greater precision in classification of individuals into a subgroup [28]. Lastly, models were selected based on interpretability, using item response probabilities to characterize and name the classes to reflect the pattern of responses [25]. LCA was conducted using MPlus 7.4 [29].

#### Sociodemographic differences in group membership

Pearson chi-square tests were used to examine whether differences in challenge group membership were related to categorical sociodemographic variables. In variables where cell sizes were small, Fisher’s exact tests were used to estimate significance. Bivariate analyses with LCA classes were conducted in Stata 15/MP [30].

#### Thematic analysis of open-ended questions

A trained research assistant coded the open-ended responses in NVivo 12 (qualitative data analysis software) using qualitative thematic analysis [31]. Following close reading of the open-ended responses, main themes were identified to develop categories. Data coded to each category were analyzed inductively to identify patterns in semantic content, develop a thematic summary of the data, and select quotes to illustrate key findings [32]. Coded data were carefully reviewed by two research team members (EL and KR), and emerging themes were discussed and refined with the research team. The qualitative analyses were completed alongside the quantitative results to expand and enrich the description of rural residents’ experiences related to the pandemic.

## Results

### Sample descriptive statistics

Surveys were completed by 279 participants (70.6% female), ranging in age from 18 to 85 (*M* = 49.5, *SD* = 14.6). Participants identified their race/ethnicity as 12.9% Indigenous, 75.3% Caucasian, and 9.7% other (e.g., Asian, south Asian, African Canadian), with 2.2% missing data. The vast majority of participants (273, 98.2%) reported having access to the internet at home, consistent with use of an online survey. Among this highly connected sample, 243 participants (87.1%), responded they had enough connected devices to meet their household needs. Most participants used computers (242, 86.7%) and smartphones (242, 86.7%), followed by tablets (157, 56.3%), voice-assistant systems (30, 10.8%), and other devices (24, 8.6%) to connect to the internet. The majority of participants (198, 71%) reported an increase in frequency of technology use to connect with family/friends and 145 (51.9%) increased frequency of technology use to gather information compared to before the onset of the pandemic. See Table 1 for a summary of participant characteristics.

### COVID challenges

Overall, 78.1% of participants reported they were highly impacted by limited access to family/friends and 76.1% reported being highly impacted by limited ability to support others. Conversely, only 20.8% reported being highly impacted by a limited access to public health information and 23.8% were highly impacted by a lack of access to daily necessities. Nearly a third (31.8%) of participants also reported being impacted by a lack of access to stable internet/mobile internet. See Table 2 for the proportion of participants reporting high impact of COVID-19 related challenges.

**Table 2:** Participant ratings of impact of challenges faced during COVID-19

	Total ***n***	% reporting high impact
**Social Needs**		
Limited access to family/friends	279	78.1
Limited ability to provide support to others	277	76.1
**Healthcare Needs**		
Limited access to healthcare services (e.g., hospital, doctor)	271	55.9
Limited access to mental health services	214	46.9
Limited access to social /support groups (e.g., addiction groups)	208	56.5
Limited access to public health information	266	20.8
**Financial Needs**		
Limited income opportunities	235	56.0
Challenges paying my bills/rent/mortgage	275	31.0
**Daily Living Needs**		
Limited access to daily necessities (e.g., food, water)	278	23.8
Limited access to options for food/grocery shopping	279	40.6
Limited access to stable internet/mobile connection	275	31.8
Limited access to childcare	128	33.9

Responses were dichotomized to indicate low impact (not at all, very little, somewhat) and high impact (quite a lot, extremely)

### LCA results

The 4-class model was the best solution that fit the data (Table 3). AIC and ABIC were all lower for the 4-class as compared to a 3-class model. Although the BIC was slightly higher for a 4-class model as compared to the 3-class model, the 4-class model had better entropy and better interpretability. We examined the pattern of responses to the challenge items across the four classes and used those to describe and assign names to the groups.

**Table 3:** Model fit information for 1 to 5 class LCA models

Classes	AIC	BIC	ABIC	BLRT	Entropy	Smallest class ***N***
1-class	3684.54	3728.07	3690.04	–	–	
2-class	3284.65	3375.34	3296.07	− 1830.27***	0.807	100
3-class	3233.55	3371.40	3250.90	− 1617.33***	0.739	75
**4-class**	**3199.21**	**3384.22**	**3222.50**	− 1578.77***	**0.821**	**40**
5-class	3176.59	3408.76	3205.82	− 1548.61***	0.829	32

*AIC* Akaike’s information criterion, *BIC* Bayesian information criterion, *ABIC* Sample size adjusted Bayesian information criterion, *BLRT* Bootstrap likelihood ratio test

*N* = 278

^***^p < 0.001

### Class descriptions

We assigned the following names to the four groups based on the pattern of challenge impacts: 1) Social Challenges (35%), 2) Social and Health Challenges (32%), 3) Social and Financial Challenges (14%), and 4) Social, Health, Financial, and Daily living Challenges (19%). The Social Challenges group had an elevated probability of experiencing high impacts related to unmet social needs, with the lowest probability of impact across daily living, healthcare, and financial needs. The Social and Health Challenges group was characterized by a high probability of experiencing impacts related to both social needs and healthcare access challenges, with lower probabilities of either financial or daily living challenges. The Social and Financial Challenges group was characterized by a high probability of both social and financial related challenge impacts, but lower probabilities of challenge impacts related to healthcare or daily living needs. Lastly, the Social, Health, Financial, and Daily Living Challenges group showed a high probability of being impacted by all COVID-related challenges examined. See Table 4 for LCA item probabilities and class membership probabilities.

**Table 4:** Class of challenges due to limited access to resources related to social, daily living, healthcare, and financial needs

Indicators		Class 1	Class 2	Class 3	Class 4
	Total Sample	Social Challenges	Social & Health Challenges	Social & Financial Challenges	Social, Health, Financial, and Daily Living Challenges
	Probability	Probability	Probability	Probability	Probability
**Social Needs**					
Limited access to family/friends	0.78	**0.54**	**0.96**	**0.75**	**0.93**
Limited ability to provide support to others	0.76	**0.51**	**0.89**	**0.89**	**0.91**
**Healthcare Needs**					
Limited access to healthcare services	0.56	0.25	**0.68**	0.43	**1.00**
Limited access to mental health services	0.47	0.12	**0.58**	0.32	**1.00**
Limited access to social /support groups	0.57	0.19	**0.79**	0.37	**1.00**
Limited access to public health information	0.21	0.03	0.21	0.08	**0.65**
**Financial Needs**					
Limited income opportunities	0.56	0.26	0.41	**1.00**	**0.96**
Challenges paying my bills/rent/mortgage	0.31	0.00	0.04	**1.00**	**0.84**
**Daily Living Needs**					
Limited access to daily necessities	0.24	0.02	0.20	0.21	**0.72**
Limited access to options for food/grocery shopping	0.41	0.12	0.44	0.38	**0.89**
Limited access to stable internet/mobile connection	0.32	0.10	0.42	0.15	**0.68**
Limited access to childcare	0.34	0.17	0.36	0.24	**0.77**
**Group Membership probability (γ)**		0.916	0.846	0.967	0.915
**Percent (*n*)**	278	35% (98)	32% (88)	14% (40)	19% (52)

Probability greater than 0.50 used to define and name groups. **Bold** indicates an elevated probability of challenge impact per indicator. For each indicator, a higher probability indicates a high challenge impact associated with a limited access to resources and needs

### Association between sociodemographic characteristics, technology use, and LCA groups

Sociodemographic characteristics and technology use were examined across the four groups (Table 5). Bivariate analyses revealed older adults (55+) were more likely to be in the Social challenges and Social and Health challenges groups, and less likely to be in Social and Financial and Social, Health, Financial, and Daily living challenges groups. Conversely, there was a higher percentage of young adults (19–35) in the Social, Health, Financial, and Daily living challenges and a higher percent of middle-aged adults (36–54) in the Social and Financial classes than expected. There were also significant differences in class by race/ethnicity (*p* = 0.005) and disability status (*p* = 0.001). In particular, there was a higher percentage of Indigenous respondents and respondents with disabilities in the Social and Health challenges and Social, Health, Financial, and Daily living challenges groups, and a higher percentage of other non-Caucasian respondents in the Social, Health, Financial, and Daily living challenges groups. There were no statistically significant differences between class membership related to gender (*p* = 0.34), education (*p* = 0.11), home type (*p* = 0.51) or ownership (*p* = 0.13), number of children in the home (*p* = 0.68), or number of seniors in the home (*p* = 0.89).

**Table 5:** Distribution of profile membership by sociodemographic characteristics and technology use

	Class 1 (35%)	Class 2 (32%)	Class 3 (14%)	Class 4 (19%)	
	Social Challenges	Social & Health Challenges	Social & Financial Challenges	Social, Health, Financial, and Daily Living Challenges	χ^**2**^ (***df***), sig
**Age**					
19–35	35.1%	24.6%	14.0%	**26.3%**	12.56(6), *p* = 0.05
36–54	28.7%	29.8%	21.3%	20.2%	
55+	41.8%	35.9%	8.7%	13.6%	
**Race/Ethnicity**^**a**^					
Indigenous	19.4%	**41.7%**	8.3%	30.6%	18.56(6), *p* = 0.01
Caucasian	38.3%	31.6%	16.3%	13.9%	
Other responses	37.0%	14.8%	11.1%	37.0%	
**Gender**					
Male	43.7%	28.2%	9.9%	18.3%	3.34(3), *p* = 0.34
Female	32.5%	33.0%	15.2%	19.3%	
**Disability**					
Yes	13.5%	**40.5%**	8.1%	**37.8%**	18.74(3), *p* < 0.001
No	39.4%	31.2%	15.5%	13.9%	
**Enough connected devices**^**a**^					
Yes	37.6%	31.0%	14.8%	16.5%	9.50(3), *p* = 0.03
No	18.8%	31.3%	12.5%	**37.5%**	
**Technology use to connect with people**					
More use	31.8%	37.4%	15.2%	15.7%	12.46(3), *p* = 0.006
Same or less use	**47.7%**	15.4%	13.9%	23.1%	
**Technology use to get information**					
More use	26.2%	**39.3%**	16.6%	17.9%	15.02(3), *p* = 0.002
Same or less use	**47.5%**	22.0%	12.7%	17.8%	

100% adds up across Classes. Total *N* = 278. Number of responses missing by sociodemographic characteristic: age (*n* = 24); gender (*n* = 10); race/ethnicity (*n* = 6); disability (*n* = 7); connected devices (*n* = 4)

^a^Due to small cell size, Fisher’s exact test were used to estimate significance level

Analyses also showed an association between reporting not having enough connected devices with being in the Social, Health, Financial, and Daily living Challenges group (*p* = 0.03). Additionally, those using technology the same or less often to connect with others or gather information during than before the pandemic were most likely to be in the Social Challenges group, whereas greater technology use to gather information was associated being in the Social and Health challenge group.

### Contextualizing Covid-19 challenges

Participants’ open-ended responses to the most significant challenge they faced during the COVID-19 pandemic paralleled the challenges described above. Similar to the LCA, social needs were a consistent challenge across participants. For example, family (*n* = 90; 32%) and social (*n* = 89; 32%) challenges were frequently described by participants as their greatest challenge. One rural-living participant described the difficulty supporting and being disconnected from an elderly family member: “*Father in law went to the hospital with a fall and now must go in a care home because of dementia and we haven’t seen him in two months*”. Challenges related to disconnection were also described by some participants as affecting their mental health. One participant, for example, wrote: “*When I could not go out to see my therapist or visit with friends it made me more of a shut-in.*” Job (*n* = 78; 28%) and financial-related (*n* = 25; 9%) challenges surrounding losing income opportunities and the inability to pay bills were described as the most significant challenge for many participants, while others described access to healthcare (*n* = 70; 25%) and daily necessities (*n* = 43; 15%) as their greatest challenges. Finally, technology-related challenges emerged as the most significant challenge during the pandemic for 12 participants (4%), as one participant described: “*Internet was always bad, but now it’s fairly useless. With kids doing school from home, they miss out on information because of our horrible internet services*”.

However, the qualitative analyses also revealed resilient outcomes not seen in the quantitative analyses. In particular, the open-ended responses from 225 participants who responded to what has been most positive about their experiences using technology during COVID-19 revolved around several themes. One quarter of the sample (*n* = 67; 24%) described technology as affording them greater convenience, such as access to meetings or healthcare, without having to travel. As one participant explained: *“The variety of different platforms to access healthcare without having to leave the house”*. A number of participants (*n* = 42; 15%) explained that technology supported social connection, as they stayed in touch with family and friends virtually. One participant described their family’s creative solution to social distancing: *“Zoom family gatherings…even game night! Feel much closer to family I can’t see in person.”* A similar number (*n* = 40; 14%) pointed to the use of technology to increase their knowledge, and stay informed during the pandemic. A smaller number of participants (*n* = 11; 4%) described the benefit of technology in terms of providing safety and options for “*staying home safe*” and avoiding unnecessary exposure to the virus. Finally, a few participants (*n* = 10; 3.6%) described their most positive experience with technology as providing leisure, or an enjoyable way to pass the time, as one participant explained: “*Keeping me occupied while at home*”.

The use of technology to manage pandemic-related challenges was evident. However, we also found that some participants had significant challenges related to technology. The most common response to participants’ biggest challenges using technology during the COVID-19 pandemic (*n* = 87, 31%) was technology issues/quality. Some participants experienced regular issues with internet quality, while others described how increased demand was slowing or even stopping internet service: *“Our internet is so slow and capped so low, we rarely are able to complete a video call or stream a movie if its not right at the beginning of the month...even then it is slow and difficult”.* Several participants (*n* = 18; 6.5%) pointed out financial issues, speaking to the need to purchase laptops for children now schooling at home and data limits being exceeded. One participant described having to make a trade-off in access of one life line for another, explaining that the biggest challenge to technology use was: *“The need to pawn my laptop and computer for food and fuel.”* A lack of digital/technology literacy meant some participants (*n* = 16; 5.7%) felt vulnerable as most everyday activities relied on digital technologies, as one participant described:*“Sometimes feeling [I] don’t have the tech knowledge to do stuff”.* Participants (*n* = 16; 5.7%) also expressed various challenges that related to the sense of loneliness and isolation, explaining that technology could not replace face-to-face interactions. The reliance on technology for social connection was described as “*less personal*” and “*depressing*”. Others (*n* = 15; 5.4%) felt burdened by “digital fatigue” or technology overuse, as one participant described: “*The burdens of increased screen time, significant eye strain and doubling up on amount of meetings.*” A minority of participants (*n* = 10; 3.6%) expressed concerns about safety and security, as well as misinformation on the internet. Others resented the fact that having the internet was an expectation, as one participant reported: *“I resent that you have to have the Internet. It is not a luxury, it is a utility”*.

## Discussion

The purpose of this study was to describe the challenges rural-living individuals faced during the early months of COVID-19, as well as explore technology use and challenges. Findings indicate that people living rurally in a Western Canadian province were most impacted by challenges related to unmet social needs and access to reliable internet in the first 4 months of the COVID-19 pandemic. Although limited access to family or friends and limited ability to support others were the highest rated challenges among survey participants, the LCA afforded us unique insights into four patterns of challenge impacts affecting different sub-sets of rural community members. Although all four groups experienced social challenges, importantly, 65% of participants also reported high levels of challenges related to daily, healthcare, or financial needs during the COVID-19 pandemic. Additionally, 1 in 5 of these participants indicated experiencing challenges in each of these areas. Recent research conducted in the United States reported negative impacts of the COVID-19 pandemic on unemployment, life satisfaction, and well-being in rural communities [33]. These authors reported the impacts were consistent across sex, education level, and race/ethnicity. In the present study, however, we found several sociodemographic differences in the types of challenges participants experienced.

Older adults experienced more challenges related to social and health needs. Although older adults are more at risk from COVID-19 in terms of mortality and hospitalization [34], it was young adults (19–25 years of age) who were impacted by a wider variety of challenges during the pandemic, which may relate to the unique milestones associated with young adulthood. Young adults may be in the process of starting a family, be less financially secure, and in need of more supports to manage their family’s needs. Additionally, middle-aged adults (36–54 years of age) were most likely to be in the Social and Financial challenges category. During middle adulthood, individuals may be caring for children and for aging parents, which may increase the impact on financial needs. Indigenous people, those with non-white ethno-racial identities, and those with a disability were also more likely to be in the Social, Health, Financial, and Daily Living challenges category compared to their Caucasian counterparts. A double jeopardy therefore exists for Indigenous and ethnic minority groups, who are both at greater risk of COVID-19 [35], and were also likely to experience a wider variety of challenges during the first wave of the pandemic. This is likely related to the existing inequities faced by these equity-denied groups.

These findings are significant because they are among the first to examine the impacts of COVID-19 challenges among subgroups of rural-living Canadians, and they support the concept that the pandemic has amplified inequity [14]. Unprecedented measures were taken to slow the spread of the virus and flatten the curve in the first wave of the pandemic when no vaccine was available; however, these measures have had disproportionate consequences for different people in rural communities [14, 35]. Historical analyses have suggested that measures to mitigate the spread of COVID-19 will have unequal consequences and economic impacts, exacerbating heath inequities [36]. The present findings provide preliminary evidence for this suggestion in rural contexts.

The rural-urban digital divide grew at the onset of COVID-19 [16], further marginalizing rural citizens. Although limited access to stable internet/mobile connection was not among the highest rated challenges, it was the most prevalent concern reported to open-ended inquire about the biggest technological challenges. It is notable that the Social, Health, Financial, and Daily Living Challenges group had the highest probability related to a lack of access to reliable internet compared to other challenge groups, suggesting that a lack of reliable internet may exacerbate other challenges. Likewise, participants’ lack of stable internet connection underscores some of the difficulty these rural-living adults had in accessing healthcare services. Indeed, COVID-19 has catalyzed a rapid massive shift to telemedicine to decrease person-to-person contact, and slow the spread of the virus [9, 37]. However, reliance on virtual connections to support healthcare has raised concerns of further health disparities and inequity for rural populations without the necessary digital infrastructure. Telemedicine used to its full capacity (e.g., video for assessment and diagnosis) requires adequate broadband access, which is often limited or unavailable in rural and underserved settings [38].

Although the vast majority of these online survey participants reported having access to the internet at home, the need and demand for internet had increased. For many, technology provided solutions to connect with family and healthcare virtually, stay informed, school children from home, and keep entertained. Yet, many rural residents that responded to this survey reported challenges related to quality, reliability, and affordability of internet and equipment even though they had access to the internet. Rural communities continue to experience complex challenges related to internet access, as only 46% of rural communities in Canada have access to high speed internet [39]. This can undermine the use of technology as a social-distancing option during a pandemic. Access is limited by broadband capacity, but also technology literacy as well as data limits and financial concerns, particularly given that there were financial and income-related challenges. Findings echo international claims that good internet access is a social determinant of health and wellbeing [40].

Overall, findings indicate that the challenges different rural community members were experiencing during the first wave of the pandemic were multidimensional and likely further exacerbated by unequal access to reliable, high speed internet. Supporting rural communities requires interventions that address localized and system-wide challenges to access to technology and essential services.

### Limitations and future research

As this was an online survey of rural community residents in a Western Canadian province primarily in the interior region, the results are not generalizable to those without internet access or residing in other areas. Additionally, this was a highly connected sample, with 98% reporting some access, and 100% having enough access to complete an online survey. This may not be reflective of most rural-living individuals. However, even among this highly connected sample, there were still key technological challenges that arose. We asked participants whether they had enough connected devices to meet the needs of their household to allow for subjective interpretation of what number of devices might be enough for different respondents; future research might include a standardized measure of number of devices to determine how many devices different rural-living respondents need. Another key limitation was the small sample size for comparative analyses, which impacted the ability to examine sociodemographic differences across challenge categories. These analyses should be replicated with a larger sample to ensure the accuracy and usefulness of the categories. That said, for the qualitative analyses this sample size was robust. The primarily female, Caucasian, who had completed trades or University education sample may not reflect the broader rural demographic; yet, important sociodemographic differences in challenges were evident. Samples including more people facing socioeconomic disadvantage would likely report greater challenges and impacts related to the costs and difficulty of access to technology.

Future research would be useful to further explore groups and communities most at risk during a pandemic, especially those isolated, living with chronic diseases, mental health or substance challenges, and older adults in long-term care. Although examining disaggregated data carries the risk of reinforcing stigmatization, doing so using a purposeful process with the intention of understanding and addressing inequities can be a force for positive change [41].

## Conclusions

Our findings provide insight about the complex and diverse needs among rural-living community members during the first wave of the COVID-19 pandemic. Taken for granted everyday activities such as grocery shopping, attending in-person medical appointments, and engaging in social interactions were moved to online platforms. As the pandemic continues, the lack of reliable internet access in rural communities will further enlarge the digital divide between the rural and urban citizens, further challenging Canada’s universal healthcare system.

The contribution of our present study is threefold. First, the survey illustrates varied sets of challenges (social, health, and financial) experienced by rural residents during the first wave of the COVID-19 pandemic. Study findings also highlight how technology use is connected to these challenges. Second, the LCA provides a new methodological approach in which to develop meaningful patterns based on citizens’ challenges. The new profiling technique offers opportunity to organize categories of citizens that experienced different forms of challenges during the COVID-19 pandemic. This type of categorization approach will be informative for policy-makers, decision-makers, and practitioners to further explore new technological solutions that address specific identified needs. Third and finally, the qualitative findings provide insights from the perspective of rural citizens during the COVID-19 pandemic.

The emerging themes presented in this study captured a diversity of areas of concern that are worth further exploration. For instance, the inter-relationship between technology literacy and concerns about misinformation and trustworthiness of online news are compounded by accessibility and affordability of technologies and the costs of multiple electronic devices and internet subscription plans. Fear of the future and pandemic restrictions of in-person interactions are exacerbating worry that rural citizens have about their adaptability. In conclusion, this research has uncovered concerning intersections of technology use, human rights and equity, and future policy planning in the context of rural communities’ access to digital technologies. More work is required at all levels of government and health and education and workplace systems to ensure reliable internet access is affordable and available to all.

## Supplementary Information

**Additional file 1.** Rural community challenges and technology use: Questionnaire Items

## Data Availability

The datasets this study is based on are stored on secure servers at The University of British Columbia - Okanagan. Anonymized data are available upon request from the corresponding author.

## Abbreviations

LCA: Latent Class Analysis
AIC: Akaike’s information criterion
BIC: Bayesian information criterion
ABIC: Sample size adjusted Bayesian information criterion
BLRT: Bootstrap likelihood ratio test

## Footnotes

1 Our definition of rural was adapted from Statistics Canada’s “rural and small town” definition18 [22].

## References

[1] Cucinotta D, Vanelli M. WHO declares COVID-19 a pandemic. Acta Biomed. 2020. 10.23750/abm.v91i1.9397. 10.23750/abm.v91i1.9397 PMC7569573 32191675

[2] Gupta M, Abdelmaksoud A, Jafferany M, et al. COVID-19 and economy. Dermatol Ther. 2020. 10.1111/dth.13329. 10.1111/dth.13329 PMC7228404 32216130

[3] Burki T. Global shortage of personal protective equipment. Lancet Infect Dis. 2020. 10.1016/S1473-3099(20)30501-6. 10.1016/S1473-3099(20)30501-6 PMC7314445 32592673

[4] Cohen J, Rodgers YVM. Contributing factors to personal protective equipment shortages during the COVID-19 pandemic. Prev Med. 2020. 10.1016/S0091-7435(20)30287-5. 10.1016/j.ypmed.2020.106263 PMC7531934 33017601

[5] Gray RS. Agriculture, transportation, and the COVID-19 crisis. Can J Agric Econ. 2020. 10.1111/cjag.12235.

[6] Castaldelli-Maia JM, Marziali ME, Lu Z, et al. Investigating the effect of national government physical distancing measures on depression and anxiety during the COVID-19 pandemic through meta-analysis and meta-regression. Psychol Med. 2021. 10.1017/S0033291721000933. 10.1017/S0033291721000933 PMC7985907 33648613

[7] Ammi M (2020). Collateral damage: the unmet health-care needs of non-COVID-19 patients.

[8] Canadian Institute for Health Information (2021). Overview: COVID-19’s impact on health care systems.

[9] Monaghesh E, Hajizadeh A. The role of telehealth during COVID-19 outbreak: a systematic review based on current evidence. BMC Public Health. 2020. 10.1186/s12889-020-09301-4. 10.1186/s12889-020-09301-4 PMC7395209 32738884

[10] Nguyen MH, Gruber J, Fuchs J, et al. Changes in digital communication during the COVID-19 global pandemic: implications for digital inequality and future research. Soc Media Soc. 2020. 10.1177/2056305120948255. 10.1177/2056305120948255 PMC7481656 34192039

[11] Desjardins PM (2011). Regional disparities in Canada: Interprovincial or urban/rural? Région et Développement n° 33.

[12] Government of Canada (2018). Public health measures: Canadian pandemic influenza preparedness: planning guidance for the health sector.

[13] Henning-Smith C, Tuttle M, Kozhimannil KB. Unequal distribution of COVID-19 risk among rural residents by race and ethnicity. J Rural Health. 2021. 10.1111/jrh.12463. 10.1111/jrh.12463 PMC7273062 32396220

[14] Canadian Human Rights Commission (2020). Statement - Inequality amplified by COVID-19 crisis.

[15] Weeden SA, Kelly W (2020). Addressing the digital divide: COVID-19 and the importance of connecting rural Canada.

[16] Canada West Foundation (2020). NEWS RELEASE | COVID-19 pandemic has exacerbated the digital divide between rural and urban communities.

[17] Canadian Internet Registration Authority (2020). New internet performance data shows urban speeds improving while rural speeds plateau.

[18] Province of British Columbia (2021). Geography of BC.

[19] Province of British Columbia (2020). BC's restart plan: Next steps to move BC through the pandemic.

[20] Province of BC (2020). Premier outlines plan to restart B.C. safely.

[21] Kotyk A (2021). Scroll through this timeline of the 1st year of COVID-19 in B.C.

[22] du Plessis V, Beshiri R, Bollma RD (2002). Definitions of rural: research paper..

[23] Statistics Canada (2018). Canadian internet use survey.

[24] Statista (2017). Number of internet connected and connectable devices in the United States from 2016 to 2020.

[25] Lanza ST, Cooper BR. Latent class analysis for developmental research. Child Dev Perspect. 2016. 10.1111/cdep.12163. 10.1111/cdep.12163 PMC6914261 31844424

[26] Morgan GB (2015). Mixed mode latent class analysis: an examination of fit index performance for classification.

[27] Nylund KL, Asparouhov T, Muthén BO (2007). Deciding on the number of classes in latent class analysis and growth mixture modeling: A Monte Carlo simulation study.

[28] Masyn KE, Little TD (2013). Latent class analysis and finite mixture modeling. The Oxford handbook of quantitative methods.

[29] Muthén L, Muthén B (2012). MPlus user’s guide.

[30] StataCorp. (2015). Stata statistical software: release 15.

[31] Elliott V. Thinking about the coding process in qualitative data analysis. Qual Rep. 2018. 10.46743/2160-3715/2018.3560.

[32] Braun V, Clarke V. Using thematic analysis in psychology. Qual Res Psychol. 2006. 10.1191/1478088706qp063oa.

[33] Mueller JT, McConnell K, Burow PB, et al. Impacts of the COVID-19 pandemic on rural America. Proc Natl Acad Sci U S A. 2021. 10.1073/pnas.2019378118. 10.1073/pnas.2019378118 PMC7817144 33328335

[34] World Health Organization (2020). WHO delivers advice and support for older people during COVID-19.

[35] Mein SA. COVID-19 and health disparities: the reality of "the great equalizer". J Gen Intern Med. 2020. 10.1007/s11606-020-05880-5. 10.1007/s11606-020-05880-5 PMC7224347 32410124

[36] Bambra C, Riordan R, Ford J, et al. The COVID-19 pandemic and health inequalities. J Epidemiol Community Health. 2020. 10.1136/jech-2020-214401. 10.1136/jech-2020-214401 PMC7298201 32535550

[37] Smith AC, Thomas E, Snoswell CL, et al. Telehealth for global emergencies: Implications for coronavirus disease 2019 (COVID-19). J Telemed Telecare. 2020. 10.1177/1357633X20916567. 10.1177/1357633X20916567 PMC7140977 32196391

[38] Hirko KA, Kerver JM, Ford S, et al. Telehealth in response to the COVID-19 pandemic: implications for rural health disparities. J Am Med Inform Assoc. 2020. 10.1093/jamia/ocaa156. 10.1093/jamia/ocaa156 PMC7337797 32589735

[39] Canadian Radio-television and Telecommunications Commission (2021). Broadband fund: closing the digital divide in Canada.

[40] American Medical Informatics Association (2017). Consider broadband access a social determinant of health.

[41] British Columbia’s Office of the Human Rights Commissioner (2020). Disaggregated demographic data collection in British Columbia: the grandmother perspective.

