# Supplementary material for: Internet access is a necessity: a latent class analysis of COVID-19 related challenges and the role of technology use among rural community residents
Source: BMC Public Health. 2022 Apr 27;22:845. doi: 10.1186/s12889-022-13254-1 (PMC9045795; doi:10.1186/s12889-022-13254-1)
Supplement: Supplementary file 1 — Additional file 1. Rural community challenges and technology use: Questionnaire Items [file 12889_2022_13254_MOESM1_ESM.docx]

*Supplemental File*

*Rural Community Challenges and Technology Use Questionnaire Items Included in Study*

**Part I: Demographic Questionnaire**

**1. Do you identify as:**

🞎 Female

🞎 Male

🞎 Other
🞎 Prefer not to disclose

**2. Age (in years):_______________**

**3. Not including yourself, how many individuals are currently living with you (enter 0 if none)?**

Senior (65+): ___

Adult (19-64): ____

Children and Youth (0-18): ____

**4. Are you living in a:**

🞎 Single-family home

🞎 Home on Farm/Ranch/Acreage

🞎 Townhouse

🞎 Duplex/Triplex/Fourplex

🞎 Apartment/Condo

🞎 Care facility (e.g., senior home)

🞎 Other, please specify: _________________

**5. Are you living in a rental or owned property?**

🞎 Rent

🞎 Own

**6. Community name:** ___________________________________

**7. What is the highest level of education you have completed?**

🞎 Some high school or less

🞎 Completed high school

🞎 Trades certification/college diploma

🞎 University degree

| **8. What ethnic background do you identify with (select all that apply)?** | |
| --- | --- |
| 🞎 First Nation  🞎 Metis  🞎 Inuit  🞎 Asian  🞎 South Asian | 🞎 Pacific Islander  🞎 Latin, South American  🞎 Caucasian  🞎 African/African-Canadian  🞎 Other; please specify: ____________ |

**Part II: COVID-19 Challenges and Technology Use Questions**

1. **Please rate the degree to which each of the following challenges have impacted you during the COVID-19 pandemic.**

|  | Not at all | Very little | Somewhat | Quit a lot | Extremely | Not applicable |
| --- | --- | --- | --- | --- | --- | --- |
| Limited access to family/friends (feeling lonely or isolated) | 1 | 2 | 3 | 4 | 5 | 🞎 |
| Limited ability to provide support to others | 1 | 2 | 3 | 4 | 5 | 🞎 |
| Limited access to daily necessities (e.g., food, water) | 1 | 2 | 3 | 4 | 5 | 🞎 |
| Limited access to options for food/ grocery shopping | 1 | 2 | 3 | 4 | 5 | 🞎 |
| Paying my bills/rent/mortgage | 1 | 2 | 3 | 4 | 5 | 🞎 |
| Limited access to stable internet/mobile connection | 1 | 2 | 3 | 4 | 5 | 🞎 |
| Limited access to mental health services | 1 | 2 | 3 | 4 | 5 | 🞎 |
| Limited access to healthcare services (e.g., hospital, doctor) | 1 | 2 | 3 | 4 | 5 | 🞎 |
| Limited access to childcare | 1 | 2 | 3 | 4 | 5 | 🞎 |
| Limited access to social groups/support groups (e.g., seniors groups, addictions support groups) | 1 | 2 | 3 | 4 | 5 | 🞎 |
| Limited income opportunities | 1 | 2 | 3 | 4 | 5 | 🞎 |
| Limited access to public health information | 1 | 2 | 3 | 4 | 5 | 🞎 |
| Other, please specify: __________ | 1 | 2 | 3 | 4 | 5 | 🞎 |

1. **Can you please tell us about the most significant challenge you have faced during the COVID-19 pandemic?____________________________________________________**
2. **a) Do you have access to the Internet at home?**

🞎 Yes – [skips to question 4]

🞎 No – [Question 3b follows]

🞎 Don't know

🞎 Prefer not to answer

1. **[If no] Why do you not have access to the Internet at home? Was it (Select all that apply):**

🞎 The cost of service or equipment

🞎 No need, no interest or no time

🞎 Lack of confidence, knowledge, skills or training

🞎 Limitation of use due to a disability

🞎 Too much objectionable material on the Internet

🞎 Confidentiality, security or privacy concerns

🞎 Limited or no access to the Internet

🞎 Other; please specify other reasons:_____________________

1. **Which of the following devices do you use to connect to the internet (select all that apply)?**

🞎 Desktop or laptop computer

🞎 Smartphone (supports basic features and allows access internet, install apps)

🞎 Tablet (i-Pad/Tablet)

🞎 Voice assistant systems (e.g., Google Home, Alexa, etc.)

🞎 Others (name of the devices______)

1. **Do you have enough connected devices to m****eet the needs of your household during the COVID-19 pandemic (e.g., online learning, work, etc.)?**

🞎 Yes

🞎 No – Please explain:__________________________________________________________________

1. **How often are you using technology (e.g., telephone, internet) to connect with friends/family and/or coworkers during the COVID-19 pandemic (ie since March 18) compared to before?**

| Far less often | Somewhat less often | About the same | Somewhat more often | Far more often | Prefer not to answer |
| --- | --- | --- | --- | --- | --- |
| 1 | 2 | 3 | 4 | 5 | 🞎 |

1. **How often are you using technology (e.g., telephone, internet) to gather news, health information, stock market, etc. during the COVID-19 pandemic (ie since March 18) compared to before?**

| Far less often | Somewhat less often | About the same | Somewhat more often | Far more often | Prefer not to answer |
| --- | --- | --- | --- | --- | --- |
| 1 | 2 | 3 | 4 | 5 | 🞎 |

1. **What has been positive about your experience using technology during the COVID-19 pandemic?**

_______________________________________________________________________________

1. **What has been your biggest challenge around using technology during the COVID-19 pandemic?**

_______________________________________________________________________________
